# FinTech regulation and banks’ risk-taking: Evidence from China

**DOI:** 10.1371/journal.pone.0311722

**Published:** 2024-10-17

**Authors:** Zhenlun Wu, Lisha Li, Bo Wang, Xiaomei Zhang

**Affiliations:** School of Finance, Southwestern University of Finance and Economics, Chengdu, Sichuan, China; Shandong University of Science and Technology, CHINA

## Abstract

By utilizing *China’s 2016 Implementation Plan for the Specific Rectification of Internet Financial Risks* as an exogenous shock, we employ a difference-in-differences identification strategy to investigate the impact of FinTech regulation on banks’ risk-taking. Our findings indicate that FinTech regulation strengthens banks’ deposit franchises and funding liquidity. As reliable and interest-rate-insensitive funding sources, higher deposit franchises weaken banks’ incentives for risk-taking. Further analysis, conducted to control for the potential interference of other policies, confirms the stable incremental effect of FinTech regulation. Moreover, we find that FinTech regulation tends to benefit banks with higher capital buffers and smaller sizes from a triple difference (difference-in-difference-in-difference) analysis. By focusing on the external effects of FinTech regulation, we aim to shed light on how regulatory gaps impact the formal financial system and highlight the importance of effectively regulating emerging financial entities.

## 1 Introduction

The rapid advancement of technology has significantly enhanced multiple industries. The convergence of digital technologies with finance has given rise to a burgeoning sector referred to as FinTech. Fintech leverages data analytics along with cutting-edge technologies such as artificial intelligence (AI), cloud computing, blockchain and quantum computing to drive digital innovation aimed at fostering growth within the financial domain [1]. Its applications span across peer-to-peer lending platforms, blockchain-based solutions for transactions management systems including equity crowdfunding mechanisms and mobile payment services [2]. The financial repression formed by government intervention is a significant factor that weakens the efficiency of China’s financial market [3]. This repression inhibits financial inclusion, whereas FinTech is a crucial means to enhance financial inclusion [4]. Consequently, the Chinese government initially encouraged the development of the FinTech sector. However, the unregulated FinTech sector has led to financial and social risks, as well as fierce competition against banks, resulting in deposit diversion issues. Therefore, this paper investigates the impact of FinTech regulation on banks while governing the economy and society. Despite extensive scholarly attention towards FinTech’s evolution, there remains limited understanding regarding its regulatory implications particularly within emerging economies.

With the deep integration of information technology into financial transactions, there has been a rapid emergence of FinTech companies. The FinTech sector continues to grow at an extraordinary pace, presenting a challenge for regulators worldwide who are eager to understand these new technologies and integrate FinTech companies into the existing regulatory framework. Regulators recognize the importance of FinTech innovation, particularly as a pathway to inclusive finance [5–8]. Consequently, they not only support and promote FinTech activities through relatively safe means, such as establishing regulatory sandboxes, but also aim to mitigate the negative byproducts of FinTech innovation, like illegal fundraising and Ponzi schemes. Therefore, there is a critical need for regulators to strike a balance between fostering FinTech innovation and ensuring financial stability, to prevent regulatory distortions.

The bank’s risk-taking primarily involves the potential for economic losses that may arise from various factors in its operations. These risks encompass not only the uncertainties stemming from external factors such as market fluctuations and policy adjustments, but also the deliberate risk exposure assumed by the bank in its decision-making process to pursue higher returns. We measure the bank’s risk-taking through the calculation of Z-score, which is consistent with the measurement method used by [9–13].

Initially, China’s regulators advocated for lax FinTech entry criteria, aiming to promote financial liberalization and address financial exclusion. However, this lenient approach led to unintended consequences. It resulted in a decrease in the price of financial licenses, thereby creating opportunities for FinTech companies to gain institutional rent and posing risks to the public. This, in turn, led to economic and social challenges, such as *Ezubao scandal*. Instead of fully capitalizing on their potential for inclusivity, the unchecked expansion of FinTech companies has led to significant negative consequences, including financial disintermediation and deposit outflows [2,14–20]. Notably, deposit outflows undermine the funding advantages of banks that depend on implicit guarantees [18], thereby weakening their capacity to attract deposits and fulfill their role in risk transformation [21–24]. In this context, we examine the impact of FinTech regulation on bank risk-taking behavior. By focusing on the external effects of FinTech regulation, we aim to shed light on how regulatory gaps impact the formal financial system and highlight the importance of effectively regulating emerging financial entities.

Utilizing the 2016 *’Implementation Plan for the Specific Rectification of Internet Financial Risks’* as an exogenous shock, we examine the impact of FinTech regulation on the risk-taking behavior of Chinese banks. Our difference-in-differences identification strategy follows [25–27], exploiting the cross-sectional variation in liability structures among banks. Initially, we focus on the impact of FinTech regulation on banks’ deposit franchises. Our findings indicate that FinTech regulation leads to increased deposit inflows. This, in turn, strengthens banks’ deposit franchises, resulting in enhanced funding advantages based on implicit guarantees, align with [27]. The size of core deposits plays a critical role in determining funding liquidity and preventing potential bank runs [23,28]. Customer deposits, as a key component of banks’ core liabilities, provide stable funding liquidity and significantly contribute to their capacity for mismatching funds [22,23]. Our empirical findings support the notion that higher deposit franchises, serving as reliable and interest-rate-insensitive funding sources, diminish banks’ incentives for risk-taking, which aligns with [29–31]. To reinforce the credibility of our findings, we conduct a series of robustness tests. These tests specifically exclude potentially confounding factors such as the capital controls introduced in 2016, the 2015 expansionary monetary policy, the interest rate liberalization reforms in 2015, and the new asset management regulations in 2018. We also conduct the parallel trend tests, placebo tests, and other robustness checks to confirm the validity of our conclusions. Moreover, we introduce a novel identification strategy to address potential deviations in identification, ensuring the robustness of our results.

Finally, following [25,32], we employ a triple difference (difference-in-difference-in-difference) identification strategy to explore the heterogeneous effects of FinTech regulation on banks’ risk-taking behaviors, considering the variables of regulatory capital pressure and bank size. Our findings are twofold: (1) Regulatory-driven deposit inflows have a more significant impact on banks with higher capital buffers. Banks under less regulatory pressure are more receptive to deposit inflows, thereby amplifying the effectiveness of the policy, (2) Regulatory-driven deposit inflows result in a more substantial reduction in risk-taking among banks with smaller sizes. These smaller banks, which are more dependent on core deposits, benefit more from the implementation of FinTech regulation. These insights highlight the positive spillover effects associated with FinTech regulation.

Our paper contributes by supplementing the understanding of the relationship between competition in the FinTech sector and bank risk. Existing literature focuses on the development of FinTech leading to regulatory arbitrage, creating competitive pressures on banks and compelling them to take on more risk. From the perspective of FinTech regulation, we explore whether the effects of FinTech regulation curb the unhealthy development of the FinTech sector, thereby mitigating bank risk-taking. This provides support for the "regulatory effectiveness" theory and offers theoretical backing for policymakers.

Our paper clarifies the relationship between bank franchise value and bank risk-taking in China. Existing literature presents competitive conclusions regarding the relationship between bank franchise value and risk-taking. By studying the relationship between FinTech regulation, bank franchise value, and risk-taking, we find that FinTech regulation strengthens bank franchise value and mitigates risk-taking. Our results support the "competition fragility" theory, showing that the reinforcement of bank franchise value in China alleviates bank risk-taking, providing a deeper understanding of the relationship between deposit franchise and risk-taking.

Our paper measures the role of individual bank heterogeneity in the impact of FinTech regulation on bank risk-taking through a triple difference method. Our research finds that higher capital buffers and smaller scales make banks more beneficial from FinTech regulation. This provides a theoretical foundation for optimizing risk-taking strategies in the banking sector.

The remainder of this paper is organized as follows. Section 2 presents the hypotheses development. Section 3 outlines the data, descriptive statistics, and identification strategy. Section 4 presents the empirical results and the robustness checks. Section 5 discusses the extensions. Finally, section 6 presents the conclusions.

## 2 Literatures review and hypothesis development

### 2.1 Literatures review

#### 2.1.1 Institutional background

On April 12, 2016, the State Council of China issued the *’Implementation Plan for the Specific Rectification of Internet Financial Risks*.’ The primary goal of this FinTech regulation is to protect the public interest, encourage compliant innovation in FinTech, prevent risks associated with FinTech, and establish a sustainable regulatory framework. The essential elements of the FinTech regulation are summarized as follows:

1. *Establishing FinTech Entry Criteria and Clarifying Operating Boundaries*: FinTech regulations increase the financial entry standards for FinTech companies. This includes requirements for registered capital and shareholder backgrounds. The aim of these provisions is to enhance the value of financial licenses and curb disorderly financial expansion. Furthermore, these regulations clearly delineate the operating boundaries between legal and illegal operations within the FinTech sector. This helps in addressing regulatory gaps and preventing regulatory arbitrage.
2. *Rectifying Vicious Competition*: FinTech regulation prohibits practices of vicious competition, such as offering excessively high returns to customers to gain market share. These provisions aim to ensure a level playing field and prevent the negative impacts of such competition. By guiding market interest rates to a reasonable range and curbing excessive investor speculation, FinTech regulation corrects market distortions and mitigates the impact of financial disintermediation due to the allure of high interest rates.
3. *Emphasizing Penetrating Supervision*: FinTech regulation underscores the importance of thorough supervision of funds to prevent illegal fundraising, Ponzi schemes, and other legal violations. These measures aim to mitigate the externalities associated with FinTech activities and protect public interests. A market clearing in the FinTech sector could trigger a return of deposits. In the case of P2P lending, regulators actively promote the orderly market clearing of this industry. In 2020, the number of Chinese P2P lending institutions decreased steadily from approximately 5,000 at its peak to zero by mid-November. The boom-and-bust history of P2P lending can be attributed to the absence of proper regulations. The closure of the P2P lending industry is expected to redirect deposits towards banks.

#### 2.1.2 Literatures review

Firstly, our research paper contributes to a growing body of literature examining the relationship between the FinTech sector and commercial banks. [5] investigates whether peer-to-peer lending acts as a substitute or complement to banks. [17] proposes that regulatory asymmetry leads to asymmetric competition, eroding banks’ ability to attract deposits. FinTech companies disrupt banks’ debt structures and reduce their core liabilities, causing banks to rely more heavily on short-term borrowing funds [2,14,16,20] argues that the emergence of FinTech companies leads to deposit outflows. [19] highlights that cities and banks with a higher exposure to FinTech experience more significant deposit outflows. we contribution to integrating regulatory sector into both the theoretical analysis and empirical research, aiming to offer a more lucid elucidation of the inherent linkage between the FinTech sector and the commercial bank sector, as well as expounding on the pivotal role played by regulatory sector. Based on the unique background in China of the distortion of the market by FinTech sector in the past years, we supplement the internal connection of the FinTech sector, the financial regulatory sector and the commercial bank sector, and explaining the role that regulatory sector play in facilitating the flow of deposits to commercial banks. We provide a theoretical basis for financial regulators and bank manager to clarify the internal relationship between financial technology, financial supervision and commercial banks. While literatures have established a connection between the evolution of FinTech and bank risk-taking, highlighting that the impact of FinTech on bank risk-taking behavior is mediated through competition and regulatory arbitrage [33,34], there is a dearth of literature examining whether bank risk-taking improves in response to regulatory policies governing the financial technology sector. Our study addresses this gap and lends support to the "regulatory effectiveness theory".

Secondly, to our knowledge, our research paper is the first empirical literature to explore the banks’ risk-taking of FinTech regulation. The existing literature on bank competition and bank risk-taking presents competing theories. On one hand, the "competition fragility" theory posits that competition diminishes the value of banks’ franchise, thereby compelling banks to take on more risk to compensate for the profit erosion caused by competition [33,35]. On the other hand, the "competition-stability" theory argues that competition lowers bank loan rates, benefiting borrowers and fostering stronger relationships between them and the banks [36].Previous literature has primarily focused on examining banks’ risk-taking behavior in relation to factors such as FinTech penetration [34], safety nets in the banking industry [37], banking supervision in international subsidiary locations [38], cultural values [32], negative interest rates [39], unconventional monetary policy [40], and institutional investor horizon [41]. We contribute to the points that there has been a lack of research on the effects of FinTech regulation on banks’ risk-taking, particularly considering the absence of regulations in the Chinese FinTech sector, where the phenomenon of "bad money driving out good money" has emerged. Therefore, by incorporating FinTech regulation into the analytical framework of banks’ risk-taking, our paper adds to the existing literature gap. China’s FinTech market offers a unique case that highlights the necessity for FinTech regulation. We present a distinctive background of China’s FinTech market, underscoring the imperative nature of regulating FinTech. We provide guidance to financial regulatory authorities on address market failures and rectify financial misalignment.

Lastly, our paper contributes to the recent body of literature that examines the relationship between banks’ deposit franchises and their risk-taking behavior. The existing literature on bank liquidity focuses on maturity transformation. The maturity transformation theory by [42] suggests that banks fund illiquid assets using unstable short-term liabilities. This implies that bank liquidity is a crucial indicator of a bank’s ability to operate healthily. The liquidity requirements theory points out that Basel III requires banks to hold substantial liquid assets to avoid liquidity crises, which can be one of the triggers of financial crises [43,27] argue that Basel III emphasizes the importance of bank liquidity, reducing funding costs for banks and enhancing their ability for maturity transformation and risk mismatching. FinTech regulation plays a role in strengthening banks’ deposit franchises and funding liquidity, but its impact on risk-taking is uncertain. One strand of the literature highlights that an increase in deposit franchises eliminates funding risks and encourages banks to take risks [28,44–47]. Another strand of the literature suggests that an expansion of deposit franchises hinders banks from taking risks [29–31,33,48]. Incorporate FinTech regulation into the research framework to study the impact of the FinTech sector on banks’ risk-taking in the form of exogenous shocks, we are able to clearly identify the causal effect between funding liquidity and banks’ risk-taking. Our findings indicate that higher deposit franchises weaken banks’ incentives for risk-taking, which aligns with the findings of [29–31,33,48]. Revised sentence: Our study contributes to a deeper understanding of the correlation between deposit franchises and risk-taking, drawing on established literature to provide both theoretical underpinning and empirical validation in support of encouraging commercial banks to enhance deposit franchises as a means of mitigating risk-taking.

### 2.2 Hypothesis development

#### 2.2.1 FinTech regulation and banks’ deposit franchise

According to the theory of regulatory evasion, financial innovation and financial regulations are constantly evolving and involve a mutual game process. Financial institutions engage in innovation to circumvent regulatory limitations. On the other hand, the public interest theory argues that regulatory gaps, resulting from factors like information asymmetry, excessive competition, and market fragility, lead to market failures that compromise the quality of financial services and the efficiency of resource allocation. Regulatory gaps also bring negative consequences for the public interests. Hence, regulators must strengthen regulatory measures to maintain financial stability [49,50] contend that lax financial regulations and excessive financial innovation inevitably lead to financial crises [51]. find that regulatory measures, such as clarifying the scope of financial business and strengthening entry barriers, help mitigate financial risk. Prudential regulation ensures that financial innovation does not undermine financial stability while creating value [52].

Bank franchises represent the net present value of future monopoly rents derived from financial entry criteria, competition restrictions, and other regulatory measures [44,45]. Bank franchises grant banks access to stable, reliable, and interest-rate-insensitive core liabilities (i.e., customer deposits), which are essential for facilitating fund mismatches [22–24]. In China, the implementation of Basel III incorporates the core liability ratio as a component of liquidity regulation indicators. A higher ratio of customer deposits to liabilities indicates more stable funding liquidity. Banks typically prefer using low-cost core liabilities to fund loans [21,23,53]. Consequently, stronger deposit franchises result in lower funding costs for banks, enabling them to engage in greater maturity mismatch and risk transformation [21–23].

The emergence of FinTech companies results in financial disintermediation, causing deposit outflows and undermining banks’ deposit franchises. Firstly, FinTech companies offer investors low-threshold, high-yield investment channels, diverting deposits away from traditional banks. According to [16], FinTech companies offer alternative investment channels that attract dispersed funds from customers seeking higher returns. This reduces the proportion of banks’ core liabilities and further elevates their funding costs. Secondly, due to regulatory asymmetry, FinTech companies are not subject to the same strict regulatory restrictions as banks when offering similar financial services, giving them a regulatory advantage that undermines banks’ deposit franchises [17]. As highlighted by [18], as household deposits progressively shift to online platforms, banks’ funding advantages based on implicit guarantees diminish. Thirdly, the presence of FinTech companies alters banks’ debt structures and increases their funding costs. To offset the loss of core liabilities, banks increasingly rely on wholesale funds [14].

FinTech regulation serves as a critical signal for prudential regulation, which helps weaken the negative externalities of FinTech innovation and triggers deposit inflows. Based on the aforementioned analysis, we propose the following hypothesis:

***Hypothesis 1*:** *FinTech regulation aims to reduce market failures and safeguard public interests*. *By guiding market expectations and the resultant deposit inflows*, *FinTech regulation strengthens banks’ deposit franchises*.

#### 2.2.2 FinTech regulation and banks’ risk-taking

According to financial intermediation theory, banks engage in risk transformation by converting risk-free deposits into risky loans [42]. As suggested by [28], banks with higher levels of deposits experience lower funding liquidity risks, as they have sufficient funds to meet debt obligations and avoid runs. The size of core deposits is crucial for determining funding liquidity and mitigating the risk of runs. Customer deposits, which represent banks’ core liabilities, serve as the primary funding liquidity for banks to carry out risk transformation. While FinTech regulation strengthens banks’ deposit franchises and funding liquidity, its impact on banks’ risk-taking remains uncertain.

One strand of literature highlights that the strengthened deposit franchises encourage banks to take on greater risks. [47] find that capital inflows expand banks’ loanable funds and potentially increase their deposit franchises and risk-taking. [45] present similar findings, demonstrating that an expansion of loanable funds is associated with a decline in loan quality and heightened risks for banks. [44] investigates the impact of Indonesia’s bank recapitalization program on lending and banks’ risk-taking, revealing that the program strengthens banks’ franchises and fosters higher risk-taking in the long run. [28] examine the relationship between funding liquidity and banks’ risk-taking, observing that banks with lower funding liquidity risk (as indicated by higher deposit ratio) tend to take on more risk. [46] study the (un)intended consequences of government bailouts, specifically focusing on Troubled Asset Relief Program (TARP) banks. They find that the most affected banks increase their credit risk-taking without a corresponding increase in profitability, suggesting an amplification of moral hazard incentives. Consequently, deposit inflows resulting from FinTech regulation may potentially increase banks’ risk-taking behavior and strengthen their moral hazard incentives.

Another body of literature suggests that the strengthened deposit franchises prevent banks from taking excessive risks. [48] demonstrate that banks with higher asset liquidity and a greater reliance on insured deposits are less vulnerable to failure. [29] analyze the evolution of banks’ funding structures before the global financial crisis and investigate the implications for financial stability. They argue that banks with weaker structural liquidity prior to the crisis were more prone to collapse. A higher franchise value lowers banks’ incentive for excessive risk-taking and mitigates moral hazard issues. Banks with greater franchise value are more likely to safeguard these values by limiting their exposure to risk [30,31] contend that a lower franchise value diminishes shareholder value, which can be eroded in the event of low asset returns, thus creating incentives for risk-taking. Hence, higher bank deposit franchises may weaken banks’ propensity to take risks.

We examine this through the lens of FinTech regulation as a natural experiment, identifying the causal relationship between funding liquidity and risk taking. we suggest that FinTech regulation mitigated regulatory arbitrage in the FinTech sector, guided market deposits back to banks, and bolstered bank deposit franchises. At the same time, higher deposit franchises reduce banks’ incentive to take risks. Based on the above analysis, we propose the following hypothesis:

***Hypothesis 2*:** *FinTech regulation strengthens banks’ deposit franchises and funding liquidity*. *As reliable and interest-rate-insensitive funding sources*, *higher deposit franchises weaken banks’ incentives for risk-taking*.

**Fig 1:**
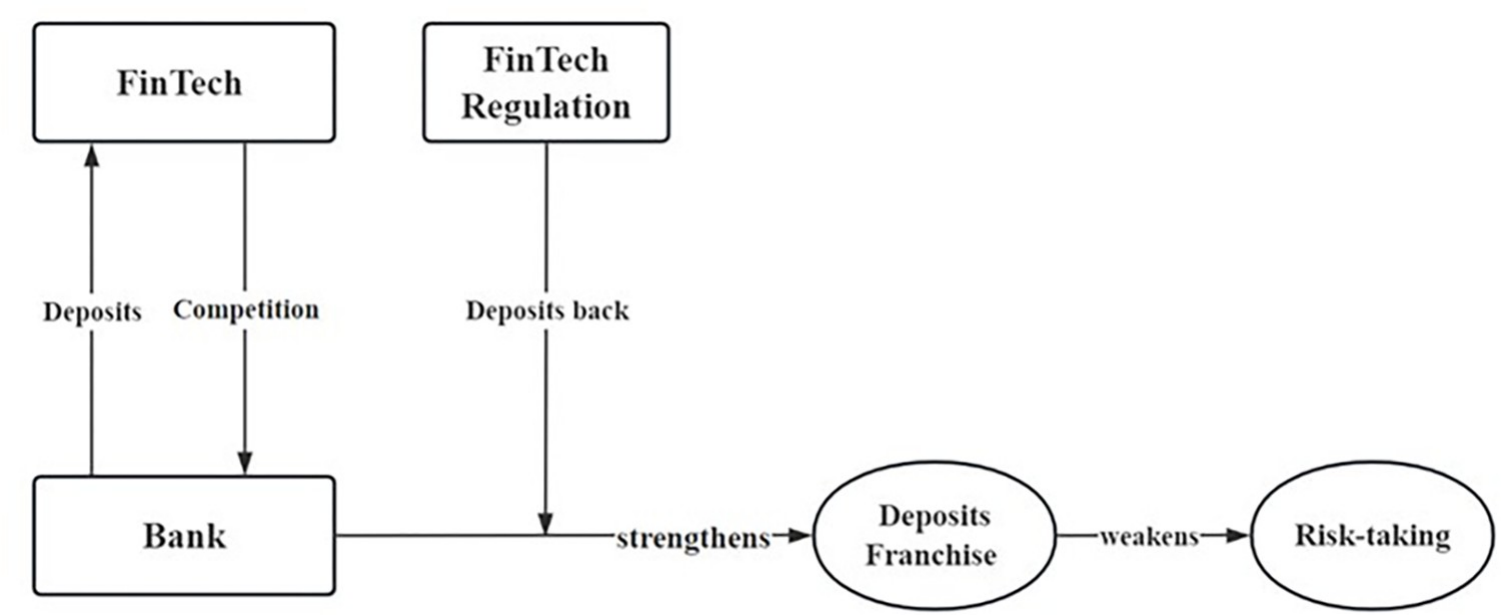
Theoretical framework.

The theoretical framework for our hypothetical development is shown below as Fig 1.

## 3 Data, descriptive statistics, and identification strategy

### 3.1 Data and descriptive statistics

We obtained Chinese commercial bank-level data from the WIND database and filled in the missing data with the CNRDS and CSMAR databases to the extent possible on a yearly basis from 2011 to 2020. We excluded bank-year data with missing information on banks’ total assets or customer deposits. We manually collated and supplemented the key and control variables. Our final dataset consists of yearly data comprises 1,990 bank-year observations for 229 unique banks. In 2015, the total assets in our dataset represented 98.77% of the total assets of Chinese commercial banks, indicating a highly representative sample for analysis (According to the China Banking and Insurance Regulatory Commission (Sources from WIND database), the total assets of commercial banks in China amounted to RMB 155,825.70 billion yuan in December 2015. In comparison, the total assets of our sample in 2015 were RMB 153,916.22 billion yuan. Our sample is highly representative since it contains approximately 98.77% of the total assets of Chinese commercial banks.). All bank balance sheet variables were winsorized at the 1st and 99th percentiles to avoid the influence of outliers.

Consistent with previous studies, we employ the Z-score as the primary measure of banks’ risk-taking. Following [13], the Z-score is calculated as the logarithm of the sum of the ratio of the average return on assets (ROA) and the equity to assets ratio (equity/assets) of a bank, divided by the standard deviation of ROA in the trailing three years. A higher value of the Z-score indicates lower risk-taking by banks. The calculation of the Z-score is as follows:

$$Z-score_{i,t}=\ln(\frac{ROA_{i,t}+Equity/A{\mathrm{ssets}}_{i,t}}{\sigma (ROA_{i,t})})$$ (1)

Table 1 presents the descriptive statistics for the key variables in the dataset. The average value of the dependent variable, Z-score, across all banks is 4.2661, with a standard deviation of 0.9438. The range of the dependent variable is substantial, with a maximum value of 6.7271 and a minimum value of 2.3584, indicating notable variations in banks’ risk-taking. The average core liabilities ratio, represented by the variable *Core_Liabilities*, is 79.5009, with a standard deviation of 12.4996. This suggests that customer deposits constitute the majority of banks’ liabilities compared to interbank deposits (*Non_Core_Liabilities*).

**Table 1:** Summary statistics.

Variables	Obs.	Mean	Std.Dev	Min	Max
Dependent Variables					
Z-score	1,526	4.2661	0.9438	2.3584	6.7271
Core_Liabilities	1,990	79.5009	12.4996	18.2331	98.7412
Non_Core_Liabilites	1,990	0.0670	0.0737	0.0000	0.3059
LLP	1,990	0.6547	0.6019	-9.6379	19.7153
*σROA*	1,544	0.1660	0.1597	0.0094	0.8516
Control Variables					
Assets	1,990	16.0435	1.7765	12.9364	21.3894
NPLR	1,990	1.6874	0.9413	0.2300	5.8500
CAR	1,990	13.7632	2.4034	8.8400	23.0700
NIIR	1,990	23.7462	21.0510	-2.1807	100.0000
ROA	1,990	1.0066	0.4513	0.0258	2.3142
Equity/Assets	1,990	7.6855	2.0179	3.7119	14.8148
Efficiency	1,990	34.1883	7.3803	18.7817	57.8784
CAR2	1,990	2.8185	2.0521	-1.8800	10.7100
Assets2	1,990	15.8279	1.7604	12.9364	21.3799

Descriptive statistics of primary variables record from 2011 to 2020 for Chinese Banks. All variables are at yearly frequency. Variables are defined in S1 Appendix.

Table 2A provides descriptive statistics of the primary variables for both treated and control banks. The last column presents the results of the t-test, showing the differences in means between treated and control banks. The t-test reveals significant differences between treated and control banks in most variables, except for the non-interest income ratio (NIIR).

**Table 2:** Summary statistics for treated banks and control banks. a. b. Summary statistics for treated banks and control banks in pre-policy period.

	Treated banks					Control banks					Treat-Control
Variables	Obs.	Mean	Std.Dev	Min	Max	Obs.	Mean	Std.Dev	Min	Max	t-test
Dependent Variables											
Z-score	521	4.4498	0.9413	2.3584	6.7271	495	4.0745	0.9212	2.3584	6.7271	0.3752***
Core_Liabilities	667	68.1152	9.6290	18.2331	98.2767	657	90.6418	6.9431	38.1930	98.7412	-22.5266***
Non_Core_Liabilites	667	0.1185	0.0809	0.0000	0.3059	657	0.0217	0.0371	0.0000	0.3059	0.0968***
Control Variables											
Assets	667	17.1118	1.6588	13.0964	20.7907	657	14.9352	1.1633	12.9364	20.8502	2.1766***
NPLR	667	1.3513	0.6878	0.2300	5.8500	657	1.9647	1.0766	0.2300	5.8500	-0.6134***
CAR	667	13.1586	2.0143	8.8400	23.0700	657	14.0935	2.6216	8.8400	23.0700	-0.9349 ***
NIIR	667	22.8177	17.3387	-2.1807	100.0000	657	22.0704	23.5585	-2.1807	100.0000	-0.7472
ROA	667	0.9397	0.3477	0.0258	2.2970	657	1.0507	0.5269	0.0258	2.3142	-0.1110***
Equity/Assets	667	6.9422	1.4128	3.7119	14.8148	657	8.2083	2.3382	3.7119	14.8148	-1.2661***
Efficiency	667	32.1342	6.9558	18.7817	56.6026	657	36.3851	7.2431	18.7817	57.8784	-4.2509***
CAR2	667	2.0965	1.3725	-0.1900	6.2300	657	3.2648	2.4128	-1.8800	10.7100	-1.1683***
Assets2	667	16.9299	1.6002	13.3605	20.2835	657	14.6882	1.1105	12.9364	20.2693	2.2417***

	Treated banks					Control banks					Treat-Control
Variables	Obs.	Mean	Std.Dev	Min	Max	Obs.	Mean	Std.Dev	Min	Max	t-test
Dependent Variables											
Z-score	187	4.1596	0.8199	2.3592	6.7271	145	4.0696	0.8077	2.3584	6.5288	0.0900
Core_Liabilities	333	70.2616	9.9506	18.2331	98.2767	305	93.5498	4.1384	70.8833	98.7412	23.2882***
Non_Core_Liabilites	333	0.1460	0.0817	0.0000	0.3059	305	0.0145	0.0248	0.0000	0.1541	0.1316***
Control Variables											
Assets	333	16.7376	1.6268	13.0964	20.3885	305	14.6256	1.1176	12.9364	20.4081	2.1121***
NPLR	333	1.0548	0.6528	0.2300	5.8500	305	1.7352	1.0263	0.2300	5.8500	-0.6804***
CAR	333	13.1103	2.2937	8.8400	23.0700	305	13.9642	2.7649	8.8400	23.0700	-0.8539 ***
NIIR	333	19.0197	14.2044	-0.9434	84.8667	305	13.1717	19.5448	-2.1807	100.0000	5.8481 ***
ROA	333	1.1247	0.3347	0.0258	2.2970	305	1.2948	0.4820	0.0258	2.3142	0.1701***
Equity/Assets	333	6.6996	1.6409	3.7119	14.8148	305	8.3954	2.4447	3.7119	14.8148	-1.6958***
Efficiency	333	32.4562	6.8945	18.7817	54.2018	305	36.1053	6.8006	20.5632	57.8784	-3.6491***
CAR2	333	2.0969	1.3777	-0.1900	6.2300	305	3.3243	2.4561	-1.8800	10.7100	-1.2275***
Assets2	333	16.9366	1.5995	13.3605	20.2835	305	14.7292	1.1291	12.9364	20.2693	2.2074***

Descriptive statistics of primary variables record from 2011 to 2020 for treated banks and control banks for Chinese Banks. All variables are at yearly frequency. Treated banks are banks in the bottom a third of the core liabilities ratio from 2013 to 2015, three years prior to FinTech regulation, while control banks are in the top a third. The last column reports the t-test difference in means between treated banks and control banks.

***, **, * indicate statistical significance at 1%, 5%, 10% respectively.

Descriptive statistics of primary variables record from 2011 to 2015 for treated banks and control banks in pre-policy period. All variables are at yearly frequency. Treated banks are banks in the bottom a third of the core liabilities ratio from 2013 to 2015, three years prior to FinTech regulation, while control banks are in the top a third. The last column reports the t-test difference in means between treated banks and control banks.

***, **, * indicate statistical significance at 1%, 5%, 10% respectively.

The Table 2B provides descriptive statistics of the primary variables for both treated and control banks in pre-policy period. The t-test shows that there is no significant difference in banks’ risk-taking variables (Z-score) between the treatment group and the control group in the pre-policy period, which also provides evidence for our parallel trend hypothesis.

### 3.2 Identification strategy

The underlying argument of our identification strategy is that banks with weak deposit competitiveness stand to benefit more from FinTech regulation. First, the expansion of FinTech companies leads to deposit outflows, and banks heavily impacted by FinTech experience more severe deposit losses [2,17,19]. Second, banks with weaker deposit franchises are more affected by FinTech developments, particularly those lagging in market shares [16,20,27,42]. This suggests that FinTech regulation, which potentially curtails some of the competitive pressures from FinTech companies, could disproportionately benefit banks that have previously struggled to maintain their deposit bases amid FinTech competition.

Banks finance themselves through core deposits to maintain liquidity, and franchise rights can enhance a bank’s ability to attract core deposits [54,55] have highlighted that the core deposit ratio serves as an indicator of a bank’s monopoly ability in the deposit market, thereby representing the value of the bank’s deposit franchise value. [56] have underscored that core deposits are a crucial gauge of a bank’s funding sources and play a pivotal role in mitigating banking fragility. [27] have established a connection between bank deposit franchise rights and core deposits by emphasizing that core liabilities are the most straightforward indicator of deposit franchise rights, which are directly influenced by the benefits derived from FinTech regulation.

Although literatures indicate that the advancement of Fintech contributes to alleviating information asymmetry and thereby reduces the risk of non-performing loans in banks [57], we focus on the impact of Fintech regulation. The 2016 Fintech regulation was designed to prohibit the Fintech sector from absorbing deposits to form a “fund pool” business and guide deposits to flow back to banks, while it did not bar banks from leveraging technological innovation to mitigate information asymmetry and prevent the issue of assets quality risks. Consequently, identifying the impact of the 2016 Fintech regulation on banks through core deposits seems reliable, yet identification through asset quality appears inconsistent with the policy setting. Likewise, the capital adequacy ratio, which is the ratio of a bank’s total capital to its risk-weighted assets, does not closely align with the primary ideology of the 2016 Fintech regulation that aims to guide deposits back to banks. It is notable that the 2016 Fintech regulation did not prohibit the financial technology sector from assisting commercial banks in alleviating the benefits brought about by information asymmetry in the realm of information technology. Instead, it curbed the Fintech sector from absorbing deposits through regulatory arbitrage to form a “fund pool” business, thereby guiding deposits to flow back and enhancing the core deposits of banks.

Our research focuses on the impact of FinTech regulation on bank risk taking. The difference-in-differences (DiD) model is a suitable econometric model for mitigating potential endogeneity issues and assessing policy effects and external shocks. It is the primary econometric model for evaluating policy effects [58]. Therefore, we employ the DiD model to examine the policy effects of FinTech regulation on commercial banks.

To examine the effect of FinTech regulation on banks’ risk-taking, we estimate the following difference-in-differences setting:

$$Y_{i,t}=\alpha_{1}+\beta_{1}(Treat_{i}\times Policy_{t})+\delta_{1}X_{i,t-1}+\gamma_{t}+\eta_{i}+\varepsilon_{i,t}$$ (2)

We define *Y*_*i*,*t*_ as the measure of risk-taking for bank *i* at time *t*. We use a dummy variable *Treat*_*i*_, which takes the value of 1 for banks in the treatment group and 0 for those in the control group. Following [26,27], we employ the core liabilities ratio to distinguish the banks. As we stated in hypothesis, the rise of FinTech has led to a diversion of funds from the market, intensifying competition within the commercial bank sector, eroding the value of bank franchise, and causing deposits to shift away from commercial banks. Simultaneously, FinTech regulation have restricted FinTech institutions (or firms)’ ability to attract funds, directing core deposits back towards banks. Consequently, prior to the implementation of FinTech regulation, banks with lower core deposit ratios benefited more from regulatory changes. We distinguished our sample as a treatment group if a bank’s core deposit ratio was in the lowest one third in 2017 and a control group if it was in the highest one third. Those in the lowest third of the core liabilities ratio distribution from 2013 to 2015 are assigned to the treatment group, while those in the highest third are in the control group. Another dummy variable, *Policy*_*t*_, is set to 1 for the period in or after 2016 and 0 otherwise. The coefficient of interest, *β*_1_, represents the average difference in risk-taking between the treatment and control groups.

*X*_*i*,*t*−1_ is a lag vector of bank *i* at time *t-1* with bank-specific characteristics to capture the cross-bank heterogeneity that may affect banks’ risk-taking over time. Among the control variables, we control for assets, non-performing loan ratio, capital adequacy ratio, noninterest income ratio, ROA, equity/assets, and efficiency. We also include year fixed effects *γ*_*t*_ to control for time-variant shocks over the sample period, which may shape banks’ risk-taking. Meanwhile, we introduce bank fixed effects *η* to control for time-invariant, unobservable bank characteristics to limit potential bias in the estimates. The regression results are correlated with bank clustering, namely, allowing the error term *ε*_*i*,*t*_ existing correlation.

Additionally, following [26,27], we employ the following continuous difference-in-differences setting for the robustness test:

$$Y_{i,t}=\alpha_{2}+\beta_{2}{\left(\frac{Core\_Liabilities}{Total\_Liabilities}\right)}_{i}\times Policy_{t}+\delta_{2}X_{i,t-1}+\gamma_{t}+\eta_{i}+\varepsilon_{i,t}$$ (3)

where ${\left(\frac{Core\_Liabilities}{Total\_Liabilities}\right)}_{i}$ is the average core liabilities ratio of bank *i* from 2013 to 2015. *β*_2_ captures our coefficient of interest. The other settings are consistent with those in Eq (2).

Due to the different settings of the treatment variables (*Treat*_*i*_ and $\left(\frac{Core_{Liablities}}{Total_{Liabilities}}\right)$) in Eqs (2) and (3), we expect that their coefficient symbol will be opposite but will capture the same policy effect. This is because the generalized difference-in-differences method applied to *Treat*_*i*_ designates banks with a lower core-liabilities ratio as the treatment group (as they are more significantly impacted by the policy). Conversely, $\left(\frac{Core\_Liablities}{Total\_Liabilities}\right)$ as a cross-sectional continuous variable in the continuous difference-in-differences method [26,27], implies that banks with a higher core-liabilities ratio are less affected by the policy. Thus, the identification mechanism of *Treat*_*i*_ is exactly opposite to that of $\left(\frac{Core\_Liablities}{Total\_Liabilities}\right)$, allowing them to capture the same policy effect through opposite regression coefficients.

## 4 Empirical results and robustness checks

### 4.1 FinTech regulation and banks’ risk-taking

Table 3 presents the impact of FinTech regulation on banks’ deposit franchises. The dependent variable in Columns (1) to (4) is the ratio of customer deposits to total liabilities (*Core_Liabilities*). Columns (1) and (2) pertain to the difference-in-differences results of Eq (2) with and without control variables, where we replace *Y*_*i*,*t*_ with banks’ *Core_Liabilities*. The coefficient of interest is the interaction term *β*_1_ between the time dummy and banks’ treatment status. With lag control variables (Column (2)), treated banks experience an increase in customer deposits to total liabilities compared to control banks, and this effect is statistically significant at the 1% level. Columns (3) and (4) present the estimated results of Eq (3), which yield similar outcomes to Columns (1) and (2).

**Table 3:** FinTech regulation and banks’ deposit franchises.

	Core_Liabilities				Non_core_liabilities			
	1	2	3	4	5	6	7	8
*Treat*_*i*_×*Policy*_*t*_	0.0147	0.0303***			-0.0701***	-0.0785***		
	(1.3631)	(2.8429)			(-10.0721)	(-11.0088)		
$(\frac{Core\_Liablities}{Total\_Liabilities}{)}_{i}\times Policy_{i}$			-0.0996**	-0.1670***			0.2778***	0.3136***
			(-2.4168)	(-4.1439)			(9.8799)	(11.0296)
Assets		-0.0656***		-0.0670***		0.0185		0.0142
		(-3.0562)		(-4.0776)		(1.4703)		(1.3112)
NPLR		-0.0003		0.0048		-0.0014		-0.0019
		(-0.0845)		(1.4584)		(-0.5771)		(-0.8659)
CAR		-0.0029		-0.0019		0.0021*		0.0016*
		(-1.5119)		(-1.2907)		(1.8941)		(1.8173)
NIIR		-0.0000		-0.0000		0.0002*		0.0003***
		(-0.0641)		(-0.2054)		(1.8382)		(2.7149)
ROA		0.0055		0.0072		-0.0132*		-0.0086
		(0.5225)		(0.8112)		(-1.8809)		(-1.4783)
Equity/Assets		0.0007		-0.0000		-0.0018		-0.0006
		(0.2190)		(-0.0178)		(-0.7971)		(-0.3449)
Efficiency		-0.0010*		-0.0005		-0.0000		-0.0001
		(-1.7171)		(-1.0813)		(-0.0216)		(-0.4075)
Bank FE	Yes	Yes	Yes	Yes	Yes	Yes	Yes	Yes
Year FE	Yes	Yes	Yes	Yes	Yes	Yes	Yes	yes
*N*	1324	1158	1990	1738	1324	1158	1990	1738
*adj*. *R*^*2*^	0.8264	0.8521	0.7732	0.8027	0.7471	0.7742	0.6767	0.7126

This table reports the impact of FinTech regulation on banks’ deposit franchises. The dependent variable in Columns (1)—(4) is customer deposits to total liabilities (Core_Liabilities), while the dependent variable in Columns (5)—(8) is interbank deposits to total liabilities (Non_Core_Liabilities). Treated banks are banks in the bottom one third of the average core liabilities ratio from 2013 to 2015, three years prior to FinTech regulation, while control banks are in the top a third. ${\left(\frac{Core\_Liabilities}{Total\_Liabilities}\right)}_{i}$ is the average ratio of customer deposits to total liabilities from 2013 to 2015. Policy is a dummy variable that takes 1 for a year in and after 2016, and 0 otherwise. Bank-level control variables include Assets, NPLR, CAR, NIIR, ROA, Equity/Assets, Efficiency. The t-statistics are based on robust standard errors clustered at the bank level in parentheses

***, **, * represent significance at 1%, 5%, 10%, respectively.

In Columns (5) to (8), the dependent variable is the ratio of interbank deposits to total liabilities (*Non_Core_Liabilities*). Columns (5) and (6) show the estimated results of Eq (2), where we replace *Y*_*i*,*t*_ with banks’ *Non_Core_Liabilities*. The interaction term *β*_1_ captures our coefficient of interest. With lag control variables (Columns (6)), treated banks exhibit a decrease in interbank deposits to total liabilities compared to control banks, and this effect is statistically significant at the 1% level. These findings indicate that FinTech regulation reduces banks’ reliance on interbank funds. Columns (7) and (8) present the estimated results of Eq (3), which show consistent outcomes with the previous columns.

Our findings suggest that FinTech regulation stimulates deposit inflows and decreases banks’ dependence on interbank funds. Consequently, FinTech regulation strengthens banks’ deposit franchises and reinforces their funding advantages based on implicit guarantees, align with [27].

Table 4 presents the impact of FinTech regulation on banks’ risk-taking. Columns (1) and (2) present the results of the difference-in-differences analysis using Eq (2). The coefficient of interest is represented by the interaction term *β*_1_. When considering lag control variables (Column (2)), compared to the control banks, the Z-scores of treated banks significantly increase, with statistical significance at the 1% level. This indicates that the implementation of FinTech regulation in 2016 effectively reduces banks’ risk-taking, suggesting external effects of FinTech regulation. Columns (3) and (4) display the estimated results of Eq (3), which yield consistent outcomes.

**Table 4:** FinTech regulation and banks’ risk-taking.

	Z-score			
	1	2	3	4
*Treat*_*i*_×*Policy*_*t*_	0.4823***	0.4831***		
	(3.3785)	(3.4288)		
${\left(\frac{Core\_Liabilities}{Total\_Liabilities}\right)}_{i}\times Policy_{t}$			-1.8279***	-1.9822***
			(-3.4657)	(-3.9305)
Assets		-0.3382		-0.4520**
		(-1.5383)		(-2.3202)
NPLR		-0.1647***		-0.1554***
		(-2.6452)		(-2.9610)
CAR		-0.0059		0.0027
		(-0.2628)		(0.1499)
NIIR		0.0016		0.0014
		(0.7666)		(0.7402)
ROA		-0.0173		-0.0910
		(-0.1225)		(-0.7589)
Equity/Assets		0.0454		0.0144
		(1.2122)		(0.4488)
Efficiency		0.0025		0.0019
		(0.3150)		(0.2453)
Bank FE	Yes	Yes	Yes	Yes
Year FE	Yes	Yes	Yes	Yes
*N*	1015	1015	1525	1525
*adj*. *R*^2^	0.4029	0.4148	0.3836	0.3936

Table 4 presents the impact of FinTech regulation on banks’ risk-taking. Treated banks are banks in the bottom one third of the average core liabilities ratio from 2013 to 2015, three years prior to FinTech regulation, while control banks are in the top a third. ${\left(\frac{Core\_Liabilities}{Total\_Liabilites}\right)}_{i}$ is the average ratio of customer deposits to total liabilities from 2013 to 2015. Policy is a dummy variable that takes 1 for a year in and after 2016, and zero otherwise. Bank-level control variables include Assets, NPLR, CAR, NIIR, ROA, Equity/Assets, Efficiency. The t-statistics are based on robust standard errors clustered at the bank level in parentheses

***, **, * represent significance at 1%, 5%, 10%, respectively.

Our findings highlight that FinTech regulation strengthens banks’ deposit franchises and funding liquidity, both of which play vital roles in enhancing their ability to mismatch funds [22,23]. As reliable funding sources, higher bank deposit franchises weaken banks’ incentives for engaging in risky activities. Our findings strongly support Hypothesis 1 and align with the conclusions drawn by [29–31,48].

### 4.2 Robustness checks

#### 4.2.1 Parallel trend test

**Fig 2:**
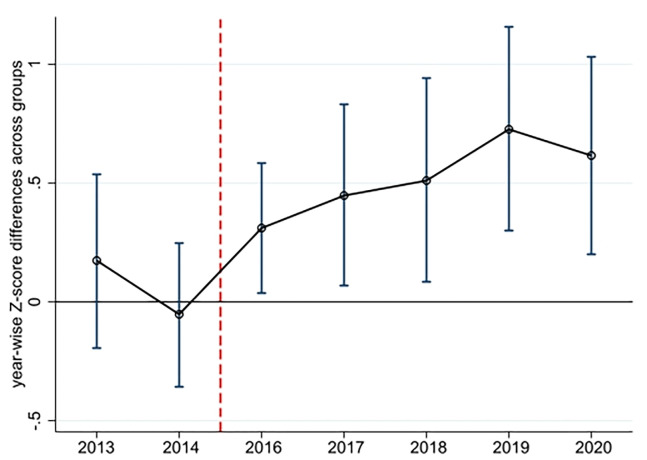
Coefficients of the parallel trend test.

The validity of our difference-in-differences approach relies on the assumption of a parallel trend, which implies that banks’ risk-taking prior to the policy implementation year is comparable between treated and control banks. To handle this assumption, we employ the parallel trend test method proposed by [59]. Fig 2 presents the diagram of the parallel trend test for banks’ Z-scores, along with 95% confidence intervals (CIs). The Z-scores are calculated using the three-year trailing method, starting from 2013. The year immediately preceding the policy implementation year (2015) is treated as the benchmark year. The broken line represents the coefficients obtained from the interaction terms between the time dummies and the banks’ treatment status, while the short vertical lines indicate the corresponding CIs.

Upon examining Fig 2, we observe that the Z-scores exhibit an upward trend and are statistically significant after 2016. However, prior to 2016, they do not exhibit statistical significance. These findings suggest that the risk-taking of the treatment groups only becomes significant after the implementation of FinTech regulation, indicating the absence of a preexisting trend.

#### 4.2.2 Placebo test

**Fig 3:**
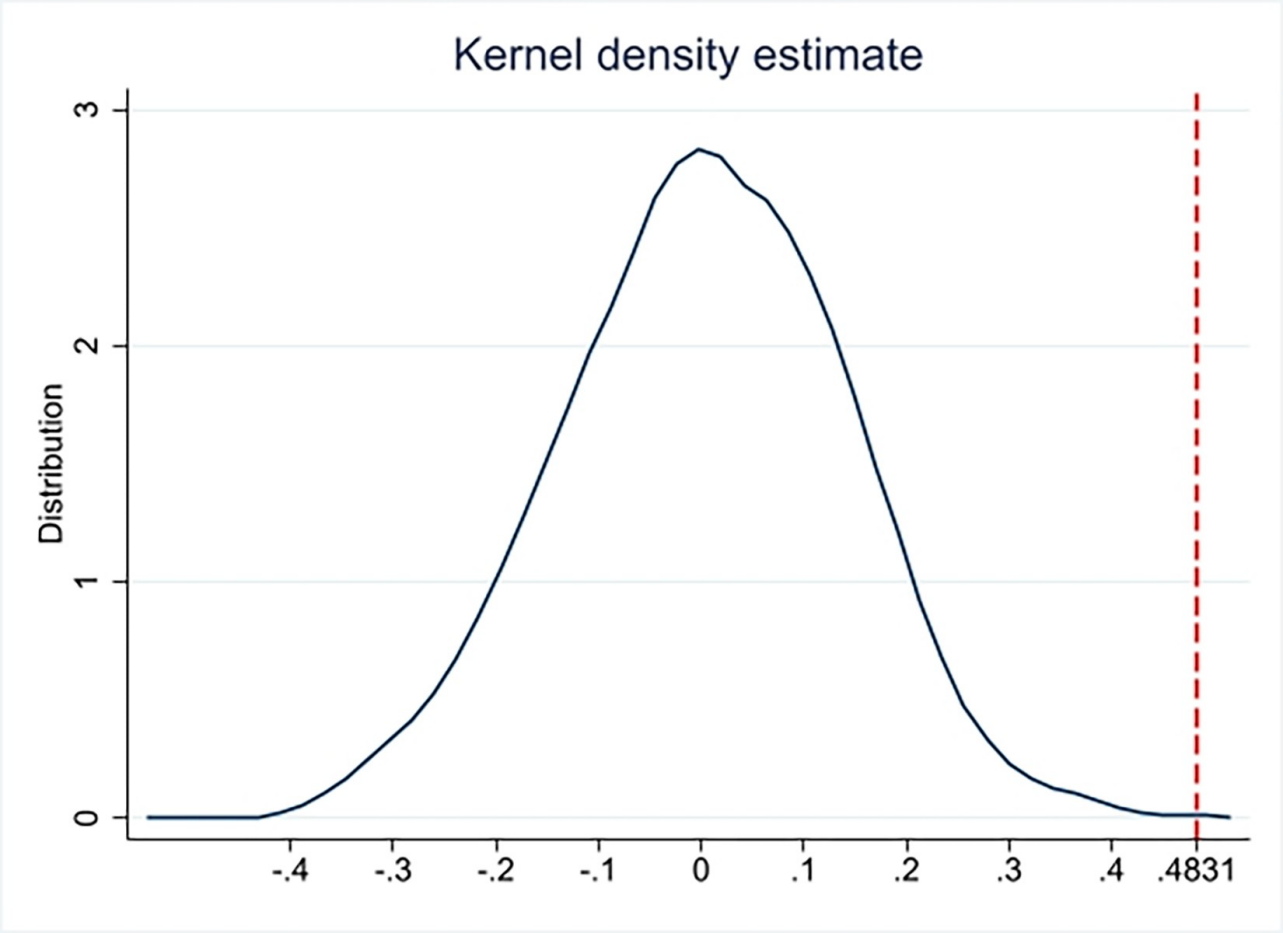
Placebo test: Random distinguish banks for 2000 times.

We follow [60] and conduct a placebo test to make the impact of FinTech regulation on banks’ risk-taking as a random effect. Specifically, we randomly assign a bank to either the treatment or control group and repeat Eq (2) 2,000 times. Fig 3 illustrates the distribution of estimated coefficients obtained from this falsification test, where the treatment and control groups are randomly assigned.

The distribution of estimates resulting from these random assignments is centered around zero, significantly lower than the benchmark estimated coefficient of 0.4831 (as shown in Column (2) of Table 4). This indicates that unobserved factors do not play a substantial role in driving the effect of FinTech regulation on banks’ risk-taking. Additionally, it suggests that any measurement error in the original treatment group is within acceptable limits.

#### 4.2.3 Alternative banks’ risk-taking variables

To provide a more comprehensive and robust analysis of the impact of FinTech regulation on banks’ risk-taking, we incorporate two additional indicators of banks’ risk-taking in our regression analysis: loan loss provision (*LLP*_*i*,*t*_) and the standard deviation of return on assets (*σROA*_*i*,*t*_). Loan loss provision (*LLP*_*i*,*t*_) serves as an indicator of banks’ prudence and preparedness to address potential credit risks in the future [61]. The standard deviation of return on assets (*σROA*_*i*,*t*_) is another important measure of banks’ risk-taking [62]. It reflects the variability or volatility of a bank’s return on assets, which is a key component of the Z-Score. In this section, we regress *LLP*_*i*,*t*_ (measured as loan loss provision over operating income) and *σROA*_*i*,*t*_ (measured as the standard deviation of return on assets over a trailing three-year period) as dependent variables in Eqs (2) and (3) to estimate the impact of FinTech regulation on banks’ risk-taking.

Table 5 presents the impact of FinTech regulation on banks’ risk-taking using alternative dependent variables. In Columns (1) and (2), we analyze the difference-in-differences results for the dependent variable *LLP*_*i*,*t*_ in Eq (2). The coefficient of interest is the interaction term β_1_. After including lag control variables (Column (2)), we find that treated banks experienced a robust decrease in *LLP*_*i*,*t*_ compared to the control group, which is statistically significant at the 5% level. In Columns (5) and (6), we examine the difference-in-differences results for the dependent variable *σROA*_*i*,*t*_ in Eq (2). The coefficient of interest is the interaction term *β*_1_. With the inclusion of lag control variables (Column (6)), we observe a significantly increase in *σROA*_*i*,*t*_ for treated banks compared to the control group, at the 1% level of significance.

**Table 5:** FinTech regulation and banks’ risk-taking: Alternative banks’ risk-taking measure.

	LLP				*σROA*			
	1	2	3	4	5	6	7	8
*Treat*_*i*_×*Policy*_*t*_	-0.1120**	-0.1583**			-0.0580**	-0.0636***		
	(-1.8917)	(-1.9680)			(-2.3810)	(-2.9249)		
${\left(\frac{Core\_Liabilities}{Total\_Liabilities}\right)}_{i}\times Policy_{t}$			0.3081	0.4228*			0.1669*	0.2281***
			(1.4999)	(1.6731)			(1.6891)	(2.7288)
Assets		-0.1941		-0.1539		0.0543		0.0834**
		(-0.9513)		(-1.0228)		(1.1116)		(2.3720)
NPLR		0.1399***		0.1319***		0.0308***		0.0275***
		(4.4365)		(5.2737)		(3.2112)		(3.1732)
CAR		-0.0144		-0.0068		-0.0033		-0.0050*
		(-0.7528)		(-0.5916)		(-0.9294)		(-1.7501)
NIIR		-0.0022		-0.0016		-0.0002		-0.0001
		(-1.6492)		(-1.3741)		(-0.4276)		(-0.4509)
ROA		-0.2148		-0.1792		0.0100		0.0124
		(-1.2559)		(-1.1984)		(0.2918)		(0.4642)
Equity/Assets		-0.0628		-0.0529		-0.0000		0.0083
		(-1.0460)		(-1.2089)		(-0.0028)		(1.3924)
Efficiency		-0.0287		-0.0197		-0.0018		-0.0016
		(-1.1989)		(-1.0283)		(-1.0147)		(-1.1335)
Bank FE	Yes	Yes	Yes	Yes	Yes	Yes	Yes	Yes
Year FE	Yes	Yes	Yes	Yes	Yes	Yes	Yes	yes
*N*	1324	1158	1990	1738	1027	1015	1544	1525
*adj*. *R*^2^	0.0897	0.4190	0.1314	0.4290	0.4940	0.5139	0.4393	0.4687

Table 5 reports the impact of FinTech regulation on banks’ risk-taking. Treated banks are banks in the bottom one third of the average core liabilities ratio from 2013 to 2015, three years prior to FinTech regulation, while control banks are in the top one third. ${\left(\frac{Core\_Liabilities}{Total\_Liabilites}\right)}_{i}$is the average ratio of customer deposits to total liabilities from 2013 to 2015. *LLP* is measured as banks’ loan-loss provision over operating income; *σROA* is measured as the standard deviation of ROA in the trailing three years. Bank-level control variables include Assets, NPLR, CAR, NIIR, ROA, Equity/Assets, Efficiency. The t-statistics are based on robust standard errors clustered at the bank level in parentheses

***, **, * represent significance at 1%, 5%, 10%, respectively.

Columns (3)—(4) and (7)—(8) report the estimated results of Eq (3) for the alternative dependent variables. The interaction term *β*_2_ captures our coefficient of interest. The estimated results indicate that our findings remain consistent and unchanged when using alternative dependent variables. Overall, the results from analyzing different dependent variables reinforce and support the main findings regarding the impact of FinTech regulation on banks’ risk-taking.

#### 4.2.4 Exclude state-owned banks and joint-stock banks

Large state-owned banks and joint-stock banks, with their extensive branch networks across China, possess stronger scale advantages, competitive edges in deposits, and more diverse funding sources compared to urban and rural banks. Furthermore, these banks may have a lower likelihood of failure due to government intervention and protection during financial distress. To mitigate potential bias arising from the risk-taking evolution being predominantly influenced by large state-owned and joint-stock banks, we exclude observations from these banks in our analysis. Additionally, it is important to note that capital outflows in 2016 led to a $320 billion reduction in China’s foreign exchange reserves, prompting authorities to implement stricter capital controls. Notably, larger banks, with their more significant international business, are more susceptible to the impact of cross-border capital flows. By excluding observations from these larger banks, we effectively isolate and address this confounding contemporaneous shock.

Table 6 illustrates the impact of FinTech regulation on banks’ risk-taking, considering the exclusion of five state-owned banks and eleven joint-stock banks (The five state-owned banks are Industrial and Commercial Bank of China, Agricultural Bank of China, Bank of China, China Construction Bank, and Bank of Communications. The 12 joint-stock banks are China Merchants Bank, Shanghai Pudong Development Bank, China CITIC Bank, China Everbright Bank, Huaxia Bank, China Minsheng Bank, China Guangfa Bank, Industrial Bank, Ping An Bank, China Zheshang Bank, Hengfeng Bank, and China Bohai Bank. However, due to senior management violations, equity confusion, poor asset quality, and tight liquidity, Hengfeng Bank failed to disclose its annual report for two consecutive years in 2017 and 2018. Therefore, we exclude Hengfeng Bank from our analysis.). Columns (1) and (2) present the results of the difference-in-differences analysis for Eq (2). The coefficient of interest is represented by the interaction term *β*_1_. When controlling for lag variables (Column (2)), the Z-scores of treated banks consistently and significantly increase compared to banks in the control group, with statistical significance at the 1% level. This suggests that the policy effect of FinTech regulation on banks’ risk-taking remains consistent regardless of bank ownership. Columns (3) and (4) show the estimated results of Eq (3), with the interaction term *β*_2_ capturing the coefficient of interest. The estimated outcomes align closely with those presented in the previous Columns (1) and (2).

**Table 6:** FinTech regulation and banks’ risk-taking: exclude state-owned banks and joint-stock banks.

	Z-score			
	1	2	3	4
*Treat*_*i*_×*Policy*_*t*_	0.4886***	0.4934***		
	(3.1862)	(3.2292)		
${\left(\frac{Core\_Liabilities}{Total\_Liabilities}\right)}_{i}\times Policy_{t}$			-1.8482***	-2.0280***
			(-3.1305)	(-3.6161)
Assets		-0.3219		-0.5663***
		(-1.3892)		(-2.8451)
NPLR		-0.1542**		-0.1497***
		(-2.4361)		(-2.8169)
CAR		-0.0022		0.0090
		(-0.0950)		(0.4989)
NIIR		0.0013		0.0011
		(0.6366)		(0.5969)
ROA		-0.0246		-0.0956
		(-0.1702)		(-0.7823)
Equity/Assets		0.0475		0.0134
		(1.2536)		(0.4169)
Efficiency		0.0009		-0.0012
		(0.1124)		(-0.1530)
Bank FE	Yes	Yes	Yes	Yes
Year FE	Yes	Yes	Yes	Yes
*N*	911	911	1389	1389
*adj*. *R*^2^	0.3784	0.3900	0.3521	0.3648

Table 6 reports the impact of FinTech regulation on banks’ risk-taking. To exclude the potential bias that the evolution of banks’ risk-taking is only driven by 5 large state-owned banks and 11 joint-stock banks, we omit the observations of these banks. Treated banks are banks in the bottom one third of the average core liabilities ratio from 2013 to 2015, three years prior to FinTech regulation, while control banks are in the top a third. ${\left(\frac{Core\_Liabilities}{Total\_Liabilites}\right)}_{i}$is the average ratio of customer deposits to total liabilities from 2013 to 2015. Policy is a dummy variable that takes 1 for a year in and after 2016, and 0 otherwise. Bank-level control variables include Assets, NPLR, CAR, NIIR, ROA, Equity/Assets, Efficiency. The t-statistics are based on robust standard errors clustered at the bank level in parentheses.

***, **, * represent significance at 1%, 5%, 10%, respectively.

#### 4.2.5 Exclude other unconventional regulatory policies

In order to prevent other unconventional policies from interfering with our focus issues, we conducted the following three tests to eliminate potential policy interference:

Firstly, The New Asset Management Regulations in 2018. The substantial growth of shadow banking in China, characterized by unregulated off-balance-sheet financial activities funded by asset management products, has contributed to the country’s debt-to-GDP ratio reaching potentially unsustainable levels. In response to this concern and to mitigate financial risks, the People’s Bank of China, along with the China Banking and Insurance Regulatory Commission, China Securities Regulatory Commission, and State Administration of Foreign Exchange, jointly issued the "Guiding Opinions on Regulating the Asset Management Business of Financial Institutions" on April 27, 2018, commonly referred to as the "New Asset Management Regulations." These regulations aim to curtail the shadow banking sector and reduce financial risk.

The New Asset Management Regulations serve two primary objectives. First, they aim to eliminate the previous practice of providing implicit guarantees of yield and principal for wealth management products offered by Chinese banks. Before these regulations, these wealth management products were often perceived as similar to deposits, owing to their guaranteed returns and capital preservation. Second, the regulations strive to regulate banks’ off-balance-sheet financial activities. This includes imposing restrictions on the issuance of high-interest wealth management products through off-balance-sheet liabilities and limiting banks’ off-balance-sheet asset business. High-yield wealth management products, while resembling deposits, are not covered by the deposit insurance system. Moreover, a significant portion of off-balance-sheet assets consists of assets that do not meet the stringent credit standards applied to banks’ on-balance-sheet assets. Consequently, the New Asset Management Regulations play a crucial role in constraining banks’ risk-taking activities. By addressing issues associated with shadow banking and introducing stricter regulations for wealth management products and off-balance-sheet activities, these regulations aim to limit banks’ exposure to risks in these areas.

Secondly, the removal of the deposit ceiling policy in 2015. In 2015, China’s regulators lifted restrictions on the deposit interest rate ceiling policy, allowing banks to break through the deposit interest rate regulation and attract deposits through higher interest rates based on individual banks’ market risk expectations and recognition. Banks that have a high interest expense to customer deposit ratio typically offer more attractive deposit yields to their customers. Such banks, which attract deposits primarily due to their competitive interest rates, are particularly sensitive to changes in deposit rate ceiling policies. Therefore, the liberalization of deposit interest rates, highlighted by the removal of these ceilings, significantly impacts these institutions. To mitigate the potential confounding effect of interest rate marketization on our conclusions, we exclude the top one-third of banks with the highest interest expense to customer deposit ratio in 2015.

Thirdly, the expansionary monetary policy in 2015. The People’s Bank of China (PBOC) implemented an expansionary monetary policy in late 2015, A reduction in the reserve requirement ratio allows banks to release more deposit funds, thus expanding their lending capacity. This increase in available funds alleviates financial burdens on banks, enhancing their liquidity and potentially influencing their risk-taking behavior. Banks with a high excess reserve ratio are often more cautious about their liquidity management. Consequently, a decrease in the reserve requirement ratio tends to have a less pronounced effect on banks with high excess reserves in terms of liquidity, compared to those with lower reserves. In essence, banks with lower excess reserve ratios benefit more in terms of liquidity enhancement from a reduction in the reserve requirement ratio. To isolate the influence of the central bank’s expansionary monetary policy on banks’ risk-taking behavior, we exclude the bottom one-third of banks based on their excess reserve to total deposits ratios in 2015. This policy can provide these banks with easier access to liquidity, potentially affecting their risk-taking propensity.

Banks may be influenced by various external policy and institutional changes. When such changes occur simultaneously with FinTech regulation, the observed outcomes may be attributed to one or more institutional changes rather than solely to FinTech regulation, leading to potential confusion in the analysis [44]. It is essential to distinguish the effects of FinTech regulation on banks’ risk-taking from the effects of other unconventional policies [63].

Table 7 shows the impact of FinTech regulation on banks’ risk taking after excluding other policies that might interfere with the baseline results.

**Table 7:** FinTech regulation and banks’ risk-taking: exclude the potential effects of other policies.

	Z-score					
	1	2	3	4	5	6
*Treat*_*i*_×*Policy*_*t*_	0.3198** (2.1909)		0.3418* (1.6668)		0.4933*** (2.7789)	
${\left(\frac{Core\_Liabilities}{Total\_Liabilities}\right)}_{i}\times Policy_{t}$		-1.7955*** (-3.3737)		-1.8979** (-2.1931)		-2.1010*** (-3.4622)
Assets	-0.2464	-0.4283*	-0.0580	-0.2905	-0.1992	-0.5286**
	(-0.8234)	(-1.7117)	(-0.2230)	(-1.2212)	(-0.7633)	(-2.2938)
NPLR	-0.0822	-0.0622	-0.0936	-0.1062	-0.1274**	-0.0864
	(-0.9733)	(-0.9386)	(-1.1233)	(-1.6027)	(-2.1581)	(-1.5679)
CAR	0.0060	0.0133	-0.0151	-0.0177	-0.0199	0.0014
	(0.2348)	(0.6894)	(-0.4997)	(-0.8082)	(-0.7410)	(0.0622)
NIIR	0.0027	0.0048*	0.0018	0.0002	0.0010	0.0028
	(0.7985)	(1.7427)	(0.7314)	(0.1112)	(0.3910)	(1.2272)
ROA	0.1560	0.1229	-0.0217	-0.0896	0.0604	0.0505
	(1.0162)	(0.9275)	(-0.1295)	(-0.6421)	(0.3850)	(0.3712)
Equity/Assets	0.0099	-0.0487	0.0896*	0.0614	0.0653	0.0140
	(0.2216)	(-1.4514)	(1.9850)	(1.6456)	(1.5467)	(0.3703)
Efficiency	-0.0031	0.0038	-0.0054	-0.0008	0.0092	0.0067
	(-0.3194)	(0.4542)	(-0.5306)	(-0.0933)	(1.0261)	(0.7669)
Bank FE	Yes	Yes	Yes	Yes	Yes	Yes
Year FE	Yes	Yes	Yes	Yes	Yes	Yes
*N*	626	938	717	1042	704	1047
*adj*. *R*^2^	0.3972	0.3799	0.4130	0.3997	0.3871	0.3898

Table 7 reports the impact of FinTech regulation on banks’ risk-taking after excluding the potential effects of other policies. Column (1)–(2) represent that exclude the potential effect of the New Asset Management Regulations by excluding the data from 2018 to 2020. Column (3)–(4) represent that exclude the potential effect of the 2015 Removal of the Deposit Ceiling Policy by excluding the upper 1/3 interest expense over customer deposits ratio in 2015. Column (5)–(6) represent that exclude the potential effect of the 2015 Expansionary Monetary Policy by excluding the lower 1/3 excess reserve to total deposits ratio in 2015. Treated banks are banks in the bottom one third of the average core liabilities ratio from 2013 to 2015, three years prior to FinTech regulation, while control banks are in the top one third. is the average ratio of customer deposits to total liabilities from 2013 to 2015. Policy is a dummy variable that takes 1 for a year in and after 2016, and 0 otherwise. Bank-level control variables include Assets, NPLR, CAR, NIIR, ROA, Equity/Assets, Efficiency. The t-statistics are based on robust standard errors clustered at the bank level in parentheses.

***, **, * represent significance at 1%, 5%, 10%, respectively.

Firstly, column (1)-(2) show the results after excluding the potential impact of the new asset management regulations. Given that the new asset management regulations were implemented in 2018, we exclude data from 2018 and later and conducted baseline regression. In column (1), the Z-scores of treated banks robustly increase compared to banks in the control group, with statistical significance at the 5% level.

Secondly, column (3)-(4) show the results of after excluding the potential impact of the removal of deposit ceiling policy. As banks with higher interest expense over customer deposits ratio are more vulnerable to the policy, we exclude the banks with the upper 1/3 interest expense over customer deposits ratio in 2015 and estimate our baseline regression. In column (3), the Z-scores of treated banks robustly increase compared to banks in the control group, with statistical significance at the 10% level.

Thirdly, Column (5)-(6) show the results of after excluding the potential impact of the expansionary monetary policy. As banks with lower excess deposit reserve over total ratio are more vulnerable to the policy, we exclude the banks with the lower 1/3 excess deposit reserve over total ratio in 2015 and estimate our baseline regression. In column (5), the Z-scores of treated banks robustly increase compared to banks in the control group, with statistical significance at the 1% level.

The results in Table 7 show that the mitigating effect of FinTech regulation on bank risk taking remains robust after excluding the potential effects of other policies.

#### 4.2.6 Alternative identification strategy

To conduct a more robust analysis of the impact of FinTech regulation on banks’ risk-taking behaviors, we introduce a novel identification strategy to address potential deviations in identification.

Firstly, we perform a regression analysis where the bank-level core liabilities ratio is regressed on the country-level market size of the FinTech industry during the pre-policy period. This regression yields a coefficient, denoted as *β*_*i*_, which represents the bank-specific sensitivity to the emergence of FinTech (referred to as Eq 4). This sensitivity coefficient is then used as a measure of a bank’s exposure to FinTech.

Next, we use these sensitivity coefficients to identify the treatment group *Fin*_*Treat*_*i*_, defined as banks with sensitivity coefficients in the bottom one-third (predominantly negative). These banks are deemed more vulnerable to FinTech industry developments, and thus, the impact of FinTech regulation is expected to be more pronounced in this group.

Finally, employing this new identification strategy (outlined in Eq 5), we analyze the effect of FinTech regulation on the risk-taking behaviors of banks. This method allows for a comprehensive assessment of the regulation’s effectiveness, taking into account the bank-specific exposure to FinTech.

$$Core\_liabilities_{i,t}=\alpha_{1}+\beta_{i}FinTech_{t}+\varepsilon_{i}$$ (4)

$$Y_{i,t}=\alpha_{1}+\beta_{1}(Fin\_Treat_{i}\times Policy_{t})+\delta_{1}X_{i,t-1}+\gamma_{t}+\eta_{i}+\varepsilon_{i,t}$$ (5)

Where *FinTech*_*t*_ represents the market size of China’s P2P industry from 2011 to 2015. Table 8 displays the results of analyzing the effect of FinTech regulation on banks’ risk-taking using this alternative identification strategy. Columns (1) and (2) present these findings. When lag control variables are included, the Z-score of the newly defined treated group shows a robust increase, which is statistically significant at the 5% level. These results are in line with our main findings, further confirming that the impact of FinTech regulation on banks’ risk-taking is consistent.

**Table 8:** FinTech regulation and banks’ risk-taking: alternative identification strategy.

	Z-score	
	1	2
*Fin*_*Treat*_*i*_×*Policy*_*t*_	0.1708	0.2994**
	(1.2099)	(2.2131)
Assets		-0.6681***
		(-2.9943)
NPLR		-0.1425***
		(-3.1305)
CAR		-0.0061
		(-0.2714)
NIIR		0.0011
		(0.5056)
ROA		0.0482
		(0.3647)
Equity/Assets		0.0527
		(1.5848)
Efficiency		-0.0061
		(-0.6603)
Bank FE	Yes	Yes
Year FE	Yes	Yes
*N*	1017	1017
*adj*. *R*^2^	0.3755	0.3967

Table 8 reports the impact of FinTech regulation on banks’ risk-taking. We place banks with the bottom one third of the sensitivity coefficient as the treatment group *Fin*_*Treat*_*i*_ (most of the sensitivity coefficients are negative), as these banks are susceptible to the development of the FinTech industry. Thus, FinTech regulation is more beneficial for the treatment group. Instead, banks with the upper one third of the sensitivity coefficient are placed in the control group. Bank-level control variables include Assets, NPLR, CAR, NIIR, ROA, Equity/Assets, Efficiency. The t-statistics are based on robust standard errors clustered at the bank level in parentheses

***, **, * represent significance at 1%, 5%, 10%, respectively.

## 5 Extensions

In this section, we deepen our analysis of the relationship between FinTech regulation and banks’ risk-taking by conducting additional tests aimed at exploring potential heterogeneous effects.

### 5.1 Regulatory capital pressure

In this section, we explore the potential heterogeneity in the effect of FinTech regulation on banks’ risk-taking, with a particular focus on regulatory capital pressure (CAR2). The interplay between capital buffers and banks’ risk-taking behaviors has been extensively examined in existing literature, primarily through two hypotheses: the regulatory hypothesis and the moral hazard hypothesis. The regulatory hypothesis posits that higher capital levels act as a buffer against risks, thereby reducing banks’ inclination towards risk-taking. Conversely, the moral hazard hypothesis suggests that banks with higher capital may exhibit increased risk-taking, driven by the belief that substantial capital levels provide a safety net for aggressive profit-seeking strategies.

Previous research offers valuable insights into this dynamic. [64] observe that banks adjust their capital buffers in response to their perceived risk exposures, with banks facing lower default risk maintaining higher capital buffers. Well-capitalized banks tend to take fewer risks, as higher capital levels offer a protective cushion against defaults. [28] argue that banks with larger capital buffers are generally more risk-averse, especially in the context of funding liquidity risk. [65] identify a positive correlation between risk levels and bank capital buffers. Meanwhile [66], report a negative, albeit not statistically significant, relationship between variations in capital buffers and banks’ risk exposure.

To assess the potential heterogeneous effects of FinTech regulation on banks’ risk-taking under varying degrees of regulatory capital pressure, we apply a difference-in-difference-in-difference framework. We measure regulatory capital pressure as the difference between a bank’s capital adequacy ratio and the minimum capital requirements. Following [25,32], we construct cross-sectional data by averaging banks’ regulatory capital pressure from 2013 to 2015. It’s important to note that systemically important banks and non-systemically important banks in China face different minimum capital requirements: 11.5% for systemically important banks and 10.5% for non-systemically important banks. To estimate the potential heterogeneous effects, we specify the following difference-in-difference-in-difference regression models.

$$\begin{array}{l} Y_{i,t}=\alpha_{3}+\beta_{31}(Treat_{i}\times Policy_{t}\times CAR2_{i})+\beta_{32}(Treat_{i}\times Policy_{t})\\ +\beta_{33}(Treat_{i}\times CAR2_{i})+\beta_{34}(Policy_{t}\times CAR2_{i})+\delta_{3}X_{i,t-1}+\gamma_{t}+\eta_{i}+\varepsilon_{i,t} \end{array}$$ (6)

$$\begin{array}{l} Y_{i,t}=\alpha_{4}+\beta_{41}\left({\left(\frac{Core\_Liabilities}{Total\_Liabilities}\right)}_{i}\times Policy_{t}\times CAR2_{i}\right)+\beta_{42}\left({\left(\frac{Core\_Liabilities}{Total\_Liabilities}\right)}_{i}\times Policy_{t}\right)\\ +\beta_{43}\left({\left(\frac{Core\_Liabilities}{Total\_Liabilities}\right)}_{i}\times CAR2_{i}\right)+\beta_{44}(Policy_{t}\times CAR2_{i})+\delta_{4}X_{i,t-1}+\gamma_{t}+\eta_{i}+\varepsilon_{i,t} \end{array}$$ (7)

where *CAP*2_*i*_ represents the cross-sectional data derived from averaging a bank’s regulatory capital pressure from 2013 to 2015. The remaining specifications are consistent with Eqs (2) and (3). The fixed effects absorb the estimated coefficients of *β*_33_ and *β*_43_. Our main focus lies in the coefficients of *β*_31_ and *β*_41_, which capture the impact of FinTech regulation on banks’ risk-taking under different levels of regulatory capital pressure.

Table 9 presents the results concerning the impact of FinTech regulation on banks’ risk-taking, taking into account variations in regulatory capital pressure. When including lag control variables (as shown in Column (2)), the estimated coefficient for *β*_31_ is 0.1317, which is statistically significant at the 10% level. This indicates that FinTech regulation has a more substantial risk-reducing effect on banks with lower levels of regulatory capital pressure. Furthermore, the significance of the estimated coefficient for *β*_41_ in Column (4) is enhanced compared to that in Column (2). Our analysis suggests that deposit inflows, spurred by regulatory measures, have a more pronounced effect in reducing risk-taking among banks with higher capital buffers. This finding implies that a lower level of regulatory pressure creates a more conducive environment for deposit inflows, thereby amplifying the effectiveness of FinTech regulation in curbing risk-taking behaviors.

**Table 9:** FinTech regulation and banks’ risk-taking: Heterogeneity effect of regulatory capital pressure.

	Z-score			
	1	2	3	4
*Treat*_*i*_×*Policy*_*t*_×*CAR*2_*i*_	0.1288	0.1317*		
	(1.6016)	(1.6590)		
${\left(\frac{Core\_Liabilities}{Total\_Liabilites}\right)}_{i}\times Policy_{t}\times CAR2_{i}$			-0.4422	-0.4305*
			(-1.6152)	(-1.7438)
*Treat*_*i*_×*Policy*_*t*_	0.1200	0.1113		
	(0.4861)	(0.4668)		
${\left(\frac{Core\_Liabilities}{Total\_Liabilites}\right)}_{i}\times Policy_{t}$			-0.5286	-0.7415
			(-0.6121)	(-0.9572)
*Policy*_*t*_×*CAR*2_*i*_	-0.0847**	-0.0915**	0.3242	0.3183
	(-2.3684)	(-2.5685)	(1.3436)	(1.4706)
Assets		-0.4175*		-0.4686**
		(-1.8980)		(-2.4605)
NPLR		-0.1712***		-0.1555***
		(-2.6995)		(-2.9613)
CAR		-0.0124		-0.0033
		(-0.5296)		(-0.1788)
NIIR		0.0016		0.0013
		(0.7901)		(0.6900)
ROA		-0.0193		-0.0876
		(-0.1368)		(-0.7304)
Equity/Assets		0.0374		0.0083
		(0.9896)		(0.2588)
Efficiency		-0.0011		-0.0003
		(-0.1366)		(-0.0447)
Bank FE	Yes	Yes	Yes	Yes
Year FE	Yes	Yes	Yes	Yes
*N*	1015	1015	1525	1525
*adj*. *R*^2^	0.4069	0.4191	0.3878	0.3970

Table 9 reports the impact of FinTech regulation on banks’ risk-taking, differentiating by regulatory capital pressure. *CAR*2_*i*_ is the cross-sectional data that generated from the average of a bank’s regulatory capital pressure from 2013 to 2015. Treated banks are banks in the bottom one third of the average core liabilities ratio from 2013 to 2015, three years prior to FinTech regulation, while control banks are in the top a third. ${\left(\frac{Core\_Liabilities}{Total\_Liabilites}\right)}_{i}$ is the average core liabilities ratio from 2013 to 2015. Policy is a dummy variable that takes 1 for a year in and after 2016, and zero otherwise. Bank-level control variables include Assets, NPLR, CAR, NIIR, ROA, Equity/Assets, Efficiency. The t-statistics are based on robust standard errors clustered at the bank level in parentheses

***, **, * represent significance at 1%, 5%, 10%, respectively.

### 5.2 Bank sizes

Another key aspect to consider is the potential heterogeneous effect of bank size (Assets2) on the interplay between FinTech regulation and banks’ risk-taking behaviors. The existing literature on this topic offers mixed conclusions. Some studies suggest that larger banks may have a lower risk of default. For example [67], argue that large banks, particularly bank-holding companies (BHCs) with diversified operations across various legal entities and geographies, can mitigate risks through business diversity. [68] examine large U.S. BHCs and conclude that the complexities arising from business, geographic, and organizational diversification can provide benefits in reducing idiosyncratic and liquidity risks.

Conversely, there are perspectives suggesting that larger banks may be riskier due to factors like moral hazards and agency problems. [31] observe a positive correlation between the geographical and business complexities of German banks and their risk-taking behavior. [46] analyze the impact of the Troubled Asset Relief Program (TARP) and note that banks participating in TARP increased their credit risk-taking without corresponding improvements in profitability. Additionally, larger banks tend to have diverse funding sources, in contrast to smaller banks, which rely more on core deposits [47]. Thus, deposit inflows driven by FinTech regulation might disproportionately benefit smaller banks.

To explore whether FinTech regulation impacts banks’ risk-taking differently based on bank size, we adopt a difference-in-difference-in-difference approach. Following [25,32], we use a bank’s total assets to create cross-sectional data, averaging the logarithm of a bank’s total assets from 2013 to 2015. We then apply the following difference-in-difference-in-difference regression models.

$$\begin{array}{l} Y_{i,t}=\alpha_{5}+\beta_{51}(Treat_{i}\times Policy_{t}\times Assets2_{i})+\beta_{52}(Treat_{i}\times Policy_{t})\\ +\beta_{53}(Treat_{i}\times Assets2_{i})+\beta_{54}(Policy_{t}\times Assets2_{i})+\delta_{5}X_{i,t-1}+\gamma_{t}+\eta_{i}+\varepsilon_{i,t} \end{array}$$ (8)

$$\begin{array}{l} Y_{i,t}=\alpha_{6}+\beta_{61}\left({\left(\frac{Core\_Liabilities}{Total\_Liabilities}\right)}_{i}\times Policy_{t}\times Assets2_{i}\right)+\beta_{62}\left({\left(\frac{Core\_Liabilities}{Total\_Liabilities}\right)}_{i}\times Policy_{t}\right)\\ +\beta_{63}\left({\left(\frac{Core\_Liabilities}{Total\_Liabilities}\right)}_{i}\times Assets2_{i}\right)+\beta_{64}(Policy_{t}\times Assets2_{i})+\delta_{6}X_{i,t-1}+\gamma_{t}+\eta_{i}+\varepsilon_{i,t} \end{array}$$ (9)

where *Assets*2_*i*_ represents the cross-sectional data derived from averaging the logarithm of a bank’s total assets from 2013 to 2015. The remaining specifications are consistent with Eqs (2) and (3). The fixed effects absorb the estimated coefficients of *β*_53_ and *β*_63_. Our main focus lies in the coefficients of *β*_51_ and *β*_61_, which capture the impact of FinTech regulation on banks’ risk-taking under different levels of bank sizes.

Table 10 illustrates the impact of FinTech regulation on banks’ risk-taking, differentiated by bank sizes. Controlling for lag variables (Column (2)), the estimated coefficient of *β*_51_ is -0.2118 and statistically significant at the 1% level, indicating that FinTech regulation has a more pronounced effect in reducing risk-taking for banks with smaller sizes. Additionally, the estimated coefficient of *β*_61_ is also significant at the 5% level (Column (4)). This suggests that deposit inflows prompted by regulatory measures have a stronger influence on reducing risk-taking among banks with smaller sizes. Smaller banks, which heavily rely on core deposits, tend to benefit more from the implementation of FinTech regulation. These findings provide further evidence of the impact and externalities associated with FinTech regulation.

**Table 10:** FinTech regulation and banks’ risk-taking: heterogeneity effect of bank sizes.

	Z-score			
	1	2	3	4
*Treat*_*i*_×*Policy*_*t*_×*Asset*2_*i*_	-0.1813**	-0.2118***		
	(-2.2690)	(-2.8758)		
${\left(\frac{Core\_Liabilities}{Total\_Liabilites}\right)}_{i}\times Policy_{t}\times Asset2_{i}$			0.5324**	0.5587**
			(2.1072)	(2.2480)
*Treat*_*i*_×*Policy*_*t*_	3.3290***	3.8390***		
	(2.6672)	(3.3198)		
${\left(\frac{Core\_Liabilities}{Total\_Liabilites}\right)}_{i}\times Policy_{t}$			-10.6593***	-11.4587***
			(-2.6436)	(-2.8973)
*Policy*_*t*_×*Asset*2_*i*_	0.1107*	0.1143*	-0.4617**	-0.5140***
	(1.7046)	(1.8615)	(-2.3121)	(-2.6554)
Assets		-0.4220*		-0.5397***
		(-1.9208)		(-2.7906)
NPLR		-0.1663***		-0.1533***
		(-2.6421)		(-2.9092)
CAR		-0.0077		0.0029
		(-0.3321)		(0.1575)
NIIR		0.0017		0.0014
		(0.8617)		(0.7275)
ROA		-0.0190		-0.0737
		(-0.1360)		(-0.6157)
Equity/Assets		0.0476		0.0197
		(1.2806)		(0.6228)
Efficiency		-0.0017		-0.0008
		(-0.2179)		(-0.1127)
Bank FE	Yes	Yes	Yes	Yes
Year FE	Yes	Yes	Yes	Yes
*N*	1015	1015	1525	1525
adj. *R*^2^	0.4054	0.4188	0.3862	0.3981

Table 10 reports the impact of FinTech regulation on banks’ risk-taking, differentiating by bank sizes. *Asset*2_*i*_ is the cross-sectional data that generated from the average logarithm of a bank’s total assets from 2013 to 2015. Treated banks are banks in the bottom one third of the average core liabilities ratio from 2013 to 2015, three years prior to FinTech regulation, while control banks are in the top one third. ${\left(\frac{Core\_Liabilities}{Total\_Liabilites}\right)}_{i}$ is the average core liabilities ratio from 2013 to 2015. Policy is a dummy variable that takes 1 for a year in and after 2016, and zero otherwise. Bank-level control variables include Assets, NPLR, CAR, NIIR, ROA, Equity/Assets, Efficiency. The t-statistics are based on robust standard errors clustered at the bank level in parentheses

***, **, * represent significance at 1%, 5%, 10%, respectively.

Table 10 highlights the effects of FinTech regulation on banks’ risk-taking behaviors, with a focus on how these effects vary by bank size. When controlling for lag variables (as shown in Column (2)), the estimated coefficient for *β*_51_ is -0.2118, which is statistically significant at the 1% level. This indicates that FinTech regulation has a more substantial impact in reducing risk-taking for smaller-sized banks. Additionally, the estimated coefficient for *β*_61_ in Column (4) is significant at the 5% level. This finding suggests that deposit inflows, driven by regulatory measures, have a stronger effect in mitigating risk-taking among smaller banks. Given their heavy reliance on core deposits, smaller banks appear to benefit more from the implementation of FinTech regulation. These results provide further evidence of the varied impacts and externalities associated with FinTech regulation.

## 6 Conclusions

This paper uses the "2016 China Internet Finance Risk Rectification Plan" as an exogenous shock and adopts the difference-in-differences identification strategy to examine the impact of FinTech regulation on bank risk-taking. Our study shows that FinTech regulation has curbed the vicious competition for market funds from China’s disorderly FinTech sector. By regulating the deposit rights of financial institutions, it emphasizes the bank franchise, particularly maintaining the licensed benefits of commercial banks’ deposit franchises, thus enhancing the bank funding liquidity. As a reliable and interest rate-insensitive source of funds, higher deposit franchise rights alleviate bank risk-taking. We conducted a series of robustness tests to confirm the stable effect of FinTech regulation. Additionally, through triple difference (difference-in-differences-in-differences) analysis, we found that FinTech regulation tends to benefit banks with higher capital buffers and smaller size, helping heterogeneous individual banks better understand how to adopt appropriate risk-taking strategies in the face of external shocks.

The empirical evaluation in this paper contributes to understanding the external effects of FinTech regulation to bank sector, and it helps to comprehend the relationship between FinTech regulation, bank franchise and bank risk-taking. By focusing on these external impacts, we aim to reveal how regulatory gaps affect the formal financial system and emphasize the importance of effectively regulating emerging financial entities. Our findings provide policy recommendations for regulators to maintain healthy development among financial institutions and curb vicious competition between different financial sectors. We also offer empirical evidence for commercial banks to adjust their risk-taking strategies in the face of external market competition and for non-bank financial institutions to clarify regulatory boundaries and develop compliant entry businesses to prevent market disruption.

Our limitations include the regional constraints of China’s FinTech regulation, making it difficult to measure the impact of the policy on economic entities in other countries. The limitation regarding our model resides in the fact that, as we have stated in the article, the 2016 Fintech regulation exerted an influence on all banks. Based on theoretical analysis and references to existing literatures, the intensity of the impact of the Fintech regulation varies among different banks, and we have designed the identification approach of the DID model through the core deposit ratio and have endeavored to guarantee the robustness of our distinguishing strategy in the robustness test. Nevertheless, our identification strategy maybe not to clearly distinguish which banks are affected by the financial technology regulation and which ones are completely unaffected. Additionally, the data sample from Chinese listed banks restricts our ability to examine the impact of FinTech regulation through capital market channels. In 2016, there were only 16 listed banks in China, making it challenging to represent the banking industry. This limitation hinders our ability to measure banks’ risk-taking through the market Z-score [69]. Moreover, bank liquidity risk is important [70], but Chinese banks disclosed liquidity risk data relatively late, thus the availability of this data also limits our ability to study the relationship between FinTech sector and bank liquidity risk. We hope future research can address these two issues. Additionally, future studies could focus on the relationship between FinTech regulation and systemic risk in banks and explore the impact of FinTech on international banks through cross-country panel data studies.

## Supporting information

S1 Appendix Table A1: Description of variables. (DOCX)
