# Supplementary material for: FinTech regulation and banks’ risk-taking: Evidence from China
Source: PLoS One. 2024 Oct 17;19(10):e0311722. doi: 10.1371/journal.pone.0311722 (PMC11486374; doi:10.1371/journal.pone.0311722)
Supplement: S1 Appendix — (DOCX) [file pone.0311722.s001.docx]

| Table A1: Description of variables. | |
| --- | --- |
| Variables | Description |
| Z-score | Z-score is the logarithm of the sum of the ratio of the average return on assets (ROA) at year t and the equity to assets ratio (Equity/Assets) at year t of a bank, divided by the standard deviation of ROA in the trailing 3-years at year t. Higher Z-scores indicate lower risk-taking. |
| Core_Liabilities | Customer deposits to total liabilities at year t. |
| Non_Core_Liabilities | Interbank deposits to total liabilities at year t. |
| Assets | The logarithm of total assets at year t. |
| NPLR | Nonperforming loans to gross loans at year t, %. |
| CAR | Capital adequacy ratio at year t, %. |
| NIIR | Non-interest income ratio at year t, %. |
| ROA | Return on assets at year t, %. |
| Equity/Assets | Equity to total assets at year t, %. |
| Efficiency | Management fees to operating revenue at year t, %. |
| LLP | Loan loss provision to operating income at year t, % |
| $\sigma ROA$ | The standard deviation of ROA in the trailing 3-years at year t. |
| CAR2 | A cross-sectional data variable generated from the average of a bank's regulatory capital pressure from 2013 to 2015, where regulatory capital pressure is defined as the capital adequacy ratio minus minimum capital requirements. The Chinese regulators stipulate that the capital adequacy ratios of systemically important banks and non-systemically important banks shall not be lower than 11.5% and 10.5%, respectively. |
| Assets2 | A cross-sectional data variable generated from the average logarithm of a bank's total assets from 2013 to 2015. |
